# Expression of CD40, CD40L and IgM production in patients with ataxia-telangiectasia

**DOI:** 10.1186/1939-4551-8-S1-A101

**Published:** 2015-04-08

**Authors:** Camila Teles Machado Pereira, Beatriz Costa Carvalho, Danielli Christinni Bichuetti-Silva, Milena Karina Coló Brunialti, Nadijane Ferreira, Reinaldo Salomão

**Affiliations:** 1 Federal University of Sao Paulo, Brazil

## Background

To examine the interaction between B and T lymphocytes through the expression of CD40 and CD40 ligand (CD40L) and IgM levels in patients with Ataxia-telangiectasia (AT).

## Methods

Blood samples were obtained from 18 AT patients (from the Federal University of Sao Paulo) and 8 age-sex-matched controls (C) (one control per day test). Total number of T, B, and NK cells were enumerated from whole blood samples using TruCount Tubes. Peripheral blood mononuclear cells (PBMC) were cryopreserved, thawed and divided in two plates, one of them with Phorbol myristate acetate (PMA) and Ionomycin to stimulate cells in vitro. After 3 hours of stimulation, cells were stained with conjugated monoclonal antibodies. In the unstimulated tube: anti-CD19-PerCP, anti-CD3-APCCy7, anti-CD8-FITC, anti-CD69-PECy7, anti-CD40-APC, anti-CD40L-PE. In the stimulated tube: anti-CD3, anti-CD8, anti-CD40, anti-CD40L. Events were analyzed by flow cytometer (BD LSRFortessa), using FlowJo Software. Linear association between IgM level and CD40 was measured via Pearson correlation. Statistical analysis was performed with SPSS 20.0 and STATA 12, and a significance threshold of <0.05 was used.

## Results

• From 18 patients, 15 were male and 3 female, aged from 5 to 25 years old. There were 3 pairs of siblings. Consanguinity was present between 2 parents. Ten of them are being treated with immunoglobulin replacement therapy. One of them had recovered from a neoplastic hematologic disease.

The total number of lymphocytes was reduced in AT patients (928 - 4579 cel/mm^3^) compared to controls (1646 – 6601 cel/mm^3^) (p=0.001). Total CD3^+^ (AT= 1163.8 cel/mm^3^; C= 2247.2 cel/mm^3^; p<0.001), CD4^+^ (AT= 531.4 cel/mm^3^; C= 1153.3 cel/mm^3^; p<0.001) and CD8^+^ (AT= 507.6 cel/mm^3^; C= 880.3 cel/mm^3^; p=0.007) numbers were decreased as well.

B cells counts also showed a reduction compared with controls (AT= 118.7 cel/mm^3^; C= 649.3 cel/mm^3^; p<0.001). By contrast, natural killer numbers were increased (AT= 583.9 cel/mm^3^; C= 357.3 cel/mm^3^; p=0.04).

Expression of CD40 was reduced compared with controls (AT= 69.9%; range: 48.6 - 94; C= 87.4%; range: 83.1 – 96; p<0.001). It wasn't found any significant statistical difference in CD40L expression between patients and controls (p=0.616), despite having stimulation documented by CD69 expression as activation marker. We found a tendency of Pearson correlation (r) between CD40 and IgM (r=0.423; p=0.091), although it wasn´t significant.

## Conclusions

Patients with AT had lower expression of CD40 on surface of B lymphocytes, which could induce abnormal production of antibodies.

